# Caffeic Acid, a Polyphenolic Micronutrient Rescues Mice Brains against Aβ-Induced Neurodegeneration and Memory Impairment

**DOI:** 10.3390/antiox12061284

**Published:** 2023-06-15

**Authors:** Amjad Khan, Jun Sung Park, Min Hwa Kang, Hyeon Jin Lee, Jawad Ali, Muhammad Tahir, Kyonghwan Choe, Myeong Ok Kim

**Affiliations:** 1 Division of Life Science and Applied Life Science (BK21 FOUR), College of Natural Sciences, Gyeongsang National University, Jinju 52828, Republic of Korea; amjadkhan@gnu.ac.kr (A.K.); jsp@gnu.ac.kr (J.S.P.); k.choe@gnu.ac.kr (K.C.); lhj4912@gnu.ac.kr (H.J.L.); jawadali666@gnu.ac.kr (J.A.); muhammadtahir30@gnu.ac.kr (M.T.); kmh1020@gnu.ac.kr (M.H.K.); 2 Department of Psychiatry and Neuropsychology, School for Mental Health and Neuroscience (MHeNs), Maastricht University, 6229ER Maastricht, The Netherlands; 3 Alz-Dementia Korea Co., Jinju 52828, Republic of Korea

**Keywords:** Alzheimer’s disease, amyloid beta, caffeic acid, neurodegeneration, antioxidants, neuroprotection, polyphenols

## Abstract

Oxidative stress plays an important role in cognitive dysfunctions and is seen in neurodegeneration and Alzheimer’s disease (AD). It has been reported that the polyphenolic compound caffeic acid possesses strong neuroprotective and antioxidant effects. The current study was conducted to investigate the therapeutic potential of caffeic acid against amyloid beta (Aβ_1–42_)-induced oxidative stress and memory impairments. Aβ_1–42_ (5 μL/5 min/mouse) was administered intracerebroventricularly (ICV) into wild-type adult mice to induce AD-like pathological changes. Caffeic acid was administered orally at 50 mg/kg/day for two weeks to AD mice. Y-maze and Morris water maze (MWM) behavior tests were conducted to assess memory and cognitive abilities. Western blot and immunofluorescence analyses were used for the biochemical analyses. The behavioral results indicated that caffeic acid administration improved spatial learning, memory, and cognitive abilities in AD mice. Reactive oxygen species (ROS) and lipid peroxidation (LPO) assays were performed and showed that the levels of ROS and LPO were markedly reduced in the caffeic acid-treated mice, as compared to Aβ-induced AD mice brains. Moreover, the expression of nuclear factor erythroid 2–related factor 2 (Nrf2) and heme oxygenase-1 (HO-1) were regulated with the administration of caffeic acid, compared to the Aβ-injected mice. Next, we checked the expression of ionized calcium-binding adaptor molecule 1 (Iba-1), glial fibrillary acidic proteins (GFAP), and other inflammatory markers in the experimental mice, which suggested enhanced expression of these markers in AD mice brains, and were reduced with caffeic acid treatment. Furthermore, caffeic acid enhanced synaptic markers in the AD mice model. Additionally, caffeic acid treatment also decreased Aβ and BACE-1 expression in the Aβ-induced AD mice model.

## 1. Introduction

Alzheimer’s disease (AD) is the most prevalent cause of dementia, which impairs memory and cognitive functions [1]. AD is characterized by two main pathological hallmarks: the accumulation of amyloid-beta (Aβ) peptide and the formation of neurofibrillary tangles (NFTs) in the brain [2]. The main enzyme responsible for the production of Aβ is β-secretase, also known as β-site amyloid precursor protein-cleaving enzyme-1 (BACE-1), which generates toxic Aβ peptide that causes AD-associated pathological changes and neurodegeneration [3,4]. Neurofibrillary tangles are formed from the hyperphosphorylation of tau, which is a microtubule-associated protein that plays a major role in the stabilization of neuronal microtubules and provides the track for intracellular transport. In the hyperphosphorylated state, the tau loses its capacity to bind to the microtubules and is unable to maintain the cytoskeleton organization, resulting in misfolded proteins and NFTs [5,6]. The accumulation of these two pathological proteins in the brain leads to oxidative stress, neuroinflammation, downregulation of the brain’s neurotrophic factors, and synaptic dysfunction [7]. Accumulation of Aβ in the brain triggers reactive oxygen species (ROS) formation and lipid peroxidation, which disrupt the intracellular defense mechanisms. The endogenous cellular antioxidant mechanism is solely regulated by certain factors, such as nuclear factor erythroid 2–related factor 2 (Nrf2) and its associated genes. Nrf2 is a major signaling pathway responsible for regulating reactive oxygen and redox signaling by activating the phase II detoxification enzymes [8,9]. Nrf2 acts as a modulator of cellular antioxidant and detoxification defense mechanisms, and its activation can reduce cellular injury and insult in several organs and tissues. Several studies have suggested that boosting the endogenous antioxidant system by upregulating the expression of Nrf2 and its downstream targets may reduce elevated oxidative stress and neuroinflammation [10,11].

The elevated oxidative stress associated with AD may induce the transcription of certain inflammatory factors such as nuclear factor kappa B (NF-κB), which is a member of mitogen-activated protein kinase. Studies have suggested that NF-κb plays an important role in the accumulation of amyloid-beta and subsequent neurodegeneration [12,13]. Apart from oxidative stress, neuroinflammation is another feature of neurodegenerative diseases executed by multiple factors, such as activated astrocytes and microglial cells. Of note, it is reported that oxidative stress is involved in the activation of astrocytes and microglial cells [14,15]. The activation of microglia and astrocytes is one of core importance for neuroinflammation and is involved in the pathogenesis of several neurodegenerative diseases, such as AD and Parkinson’s disease (PD) [16,17]. Several brain regions are susceptible to AD-related pathology; the most important one is the hippocampus, which is affected in the earlier stage of AD. Hippocampus plays a role in the storage of long- and short-term memories. Previous studies have extensively highlighted the roles of growth factors and phosphatidylinositol 3-kinases (PI3K)/protein kinase B (AKT) signaling pathways in hippocampal plasticity and memory functions. The PI3K/AKT signaling pathway promotes cell survival, proliferation, and differentiation, which are important for normal cellular activities [18,19]. Brain-derived neurotrophic factor (BDNF) is considered the key factor in the homeostasis of brain physiology and functions. This growth factor support neuronal survival, synaptic functions, and induced hippocampal neurogenesis and improve cognition [20,21]. A growing body of research shows that activations of neurotrophic factors considerably reduce the effects of oxidative stress and neuroinflammation [22,23]. 

**Figure 1:**
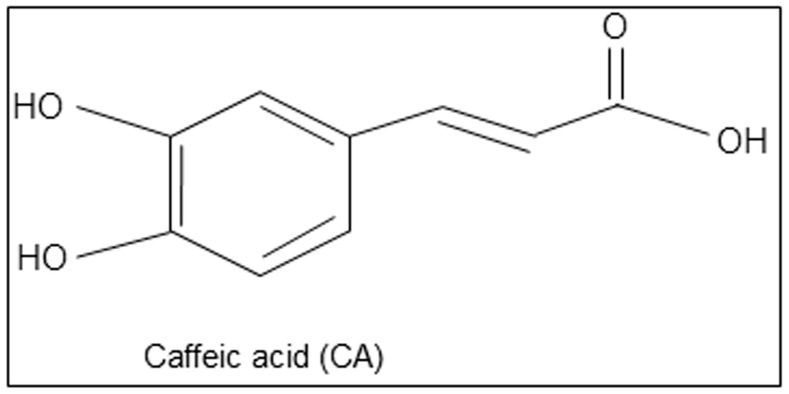
Chemical structure of caffeic acid.

Currently, there is no known treatment available to cure AD, only certain drugs may reduce the sign and symptoms associated with AD. Meanwhile, several natural compounds such as flavonoids, vitamins, phenolic acids, and polyphenols, have received special interest in the management of these diseases. The natural compounds possess antioxidant and anti-inflammatory activities and they also increase synaptic integrity, memory, and cognitive functions [24,25]. Phenolic compounds, which possess antioxidant, anticancer, antibacterial, and anti-inflammatory properties, are a group of compounds mainly found in fruits and vegetables [26,27]. Here, we hypothesize that caffeic acid, a polyphenolic compound found in vegetables, fruits, and herbs may reduce AD symptoms and its pathological features. The chemical structure of caffeic acid is given in Figure 1. 

## 2. Materials and Methods

### 2.1. Chemicals

Caffeic acid (CAS 331-39-5), amyloid beta-peptide (Aβ), and all the primary antibodies were obtained from Santa Cruz Biotechnology, (Dallas, TX, USA). Table 1 lists the primary antibodies used in the study. 

### 2.2. Intracerebral Injection Amyloid-Beta and Mice Grouping

Stock solution (1 mg/mL) of the human Aβ_1–42_ peptide was prepared in sterile saline, and incubated at 37 °C for four days to aggregate the peptide. Mice were anesthetized by ketamine/xylene and placed in a stereotaxic frame; the bregma point of the skull was exposed. Aβ_1–42_ (5 μL/5 min/mouse) was injected intracerebroventricularly (i.c.v) by using a Hamilton syringe (Trajan Scientific and Medical, Victoria Australia). The coordinates were adjusted from the bregma: 2.4 mm dorsoventral (DV), 0.2 mm anteroposterior (AP), and 1 mm mediolateral (ML). The body temperature was maintained with a heating pad.

Randomly, the mice were divided into four groups (*n* = 8 mice/group): 1—control group (normal mice treated with saline), 2—AD-induced mice (mice that received Aβ_1–42_ i.c.v); 3—treated group of mice (AD mice treated with caffeic acid, orally for two weeks at a dose of 50 mg/kg/day); 4—normal mice treated with caffeic acid for two weeks (50 mg/kg/day). The four groups of mice used in the study are identified as: 1. Cont; 2. CA; 3. Aβ; and 4. Aβ + CA. All the experiments involving animals were implemented in agreement with guidelines and principles approved by the Institutional Animal Care and Use Committee (IACUC), Division of Applied Life Sciences, Gyeongsang National University, South Korea (Approval ID: 125).

### 2.3. Behavior Study

After completion of the drug treatments, the mice were adjusted for two days and then the Y-maze test and Morris water maze test (MWM) behavioral studies were conducted. 

#### 2.3.1. Y-Maze Test

The Y-maze apparatus was made from transparent plastic and had three arms, which were oriented at a 120° angle from each other. The arms had a 20 cm height, 10 cm width, and 50 cm length. The mice were put in the middle of the maze and allowed to explore its three arms for eight minutes. The movement of the mice in the maze was observed. Spontaneous alternation was defined as the entry of the rodents into the alternate arm, while the total number of arm entries was also considered. The alteration behavior percentage (%) was calculated using the formula: entries into three arms consecutively/total number of arm entries −2 × 100. A higher percentage of spontaneous alternation behavior was considered to indicate enhanced spatial working memory.

#### 2.3.2. Morris Water Maze Test

The MWM test is conducted in a circular tank that is 100 cm in diameter and 40 cm in height. The tank was filled with opaque water to a depth of 15.5 cm, at room temperature (23 ± 0.5 °C). A hidden platform was placed in one quadrant, 1 cm below the water surface. The platform remained in the same position during the training session and was removed from the probe test. The MWM was performed as described previously [28,29]. All mice were trained for five days and the latency was considered for each trial to reach the hidden platform. After the training was finished, the probe test was carried out by removing the hidden platform. The mice were allowed to swim freely in the water tank for 1 minute. The number of crossings and time spent in the target quadrant were analyzed. The data were recorded using video-tracking software (SMART V3.0 Panlab Harvard Apparatus Bioscience Company, Holliston, MA, USA). 

### 2.4. Protein Extraction for Western Blot and Biochemical Analysis

After completion of the behavioral studies, the mice were randomly divided into two groups, one for Western blot and biochemical analysis and the other for immunofluorescence studies. For the Western blotting and biochemical assays, the mice were anesthetized (with ketamine/xylene), the brains were removed, and the hippocampus was carefully dissected. The hippocampal tissues were homogenized in PRO-PREP^TM^ extraction solution (iNtRON Biotechnology, Inc., Sungnam, Republic of Korea). The supernatant was collected and kept at −80 °C for experimentation after being centrifuged at 13,000 revolutions per minute for 25–30 minutes at 4 °C.

### 2.5. Collection of Brains for Morphological Analysis

As mentioned previously [30,31,32], the mice were anesthetized and perfused transcardially with 0.9% normal saline and 4% paraformaldehyde solution. The brains were carefully removed and fixed in ice-cold 4% neutral buffer paraformaldehyde (4 °C for 72 h). The brains were dehydrated in 20% sucrose for 72 h after fixation. The brains were placed in an optimum cutting temperature (O.C.T.) compound obtained from Finetek Japan C., Ltd., Tokyo, Japan, and then frozen. By using a microtome (Leica cryostat CM 3050S, Nussloch, Germany), 14 µm sections were obtained on gelatin-coated slides.

### 2.6. Western Blotting

Western blot was performed as conducted previously [33,34,35,36]. The concentration of proteins was measured with a Bio-Rad protein assay kit (Bio-Rad Laboratories, Hercules, CA, USA). An equal amount of proteins were loaded in the SDS-PAGE (12–15%) gel with a prestained protein marker (GangNam-STAIN, iNtRON Biotechnology, Dallas, TX, USA) and transferred to polyvinylidene-difluoride (PVDF) membranes (Immobilon-PSQ, transfer membrane, Merck Millipore, Burlington, MA, USA). After blocking with 5% skim milk, the membranes were incubated at 4 °C with primary antibodies. After incubation, the membranes reacted with HRP (horseradish peroxidase)-conjugated secondary antibodies. The fluorescence was visualized using ECL (enhanced chemiluminescent) detection reagent (ATTO Corporation, Tokyo, Japan). The expressions of the bands were detected on the X-ray film. For densitometric analysis, ImageJ software (v.1.50, NIH, Bethesda, MD, USA) was used.

### 2.7. Immunofluorescence Analysis 

Immunofluorescence was performed as described previously [37,38,39]. Slides were washed with 1% phosphate-buffered saline (PBS) and reacted with Proteinase-k for 5 minutes; next, a blocking solution containing normal serum 2% and 0.3% Triton X-100 in 1% PBS was incubated. Primary antibodies were applied to the slides overnight at 4 °C, followed by washing. After that, the slides were reacted with TRITC (tetramethylrhodamine isothiocyanate) or FITC (fluorescein isothiocyanate)-labeled secondary antibodies (Santa Cruz Biotechnology, Dallas, TX, USA). After secondary antibody treatment, the slides were reacted with DAPI (4, 6-diamidino-2-phenylindole) for nuclear staining. The slides were covered with a coverslip using a fluorescence mounting medium. A confocal microscope (FluoView FV 1000; Olympus, Tokyo, Japan) was used to take the images. 

### 2.8. Reactive Oxygen Species (ROS) Assay

Hippocampal brain homogenate was diluted to obtain the final concentration of 5 mg of the tissue/mL. A solution containing 1 mL Lock’s buffer (pH ± 7.4) and 0.2 mL of homogenates was mixed with 10 mL of 5 mm 7-dichlorodihydrofluorescein diacetate (DCFH-DA). The mixture was incubated at room temperature to form fluorescent DCF. A microplate reader at 484 nm (excitation) and 530 nm (emission) was used to monitor the conversion of DCF from DCHFH-DA. Parallel blanks were used to control the absorbance in the absence of homogenate (background fluorescence). The ROS is expressed as DCF formed (pmol)/amount of protein (mg).

### 2.9. Lipid Peroxidation (LPO) Assay

The LPO experiment was carried out in accordance with the assay kit’s instructions (catalog # K739-100 Biovision Incorporated, Milpitas, CA, USA); the LPO was examined in the hippocampus of experimental animals. Malondialdehyde (MDA), a biomarker of LPO, was measured utilizing the thiobarbituric acid reactive substance (TBARS) to determine the presence of LPO. Standard TEP (1, 1, 3, 3-tetra ethoxy propane) was used to measure the TBARS content by using a spectrophotometer (absorbance at 535 and 520 nm), and the data were evaluated as relative MDA nmol/mg protein. 

### 2.10. Evaluations and Statistical Analysis

For densitometric analysis of Western blotting and immunofluorescence analysis, we used ImageJ software. For the preparation of graphs and statistical analysis, we used GraphPad Prism 8 (GraphPad Software Inc., San Diego, CA, USA). Behavior results were obtained from 8 mice/group. For Western blot and confocal microscopy, the data were obtained from 4 mice/group respectively and are representative of three experiments. Results are presented as a mean ± standard deviation and analyzed by one-way ANOVA followed by Tukey’s multiple comparisons tests. A value of *p* < 0.05 was considered significant.

## 3. Results

### 3.1. Caffeic Acid Improved Memory Impairments and Inhibited Aβ and BACE-1 Protein Expression in AD Mice Brain

**Figure 2:**
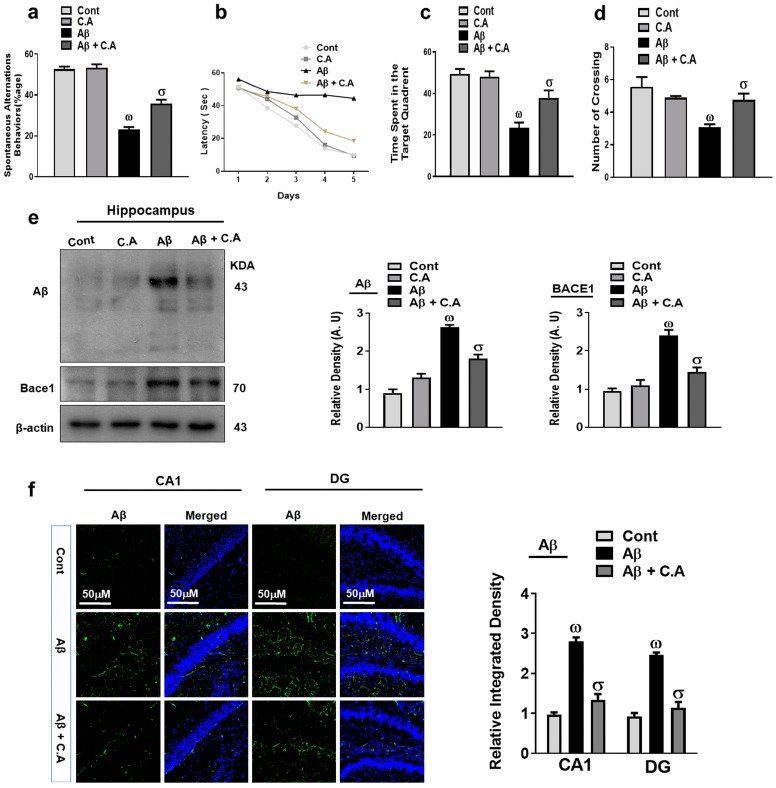
Neuroprotective effect of caffeic acid on memory functions Aβ and BACE-1 expression. (**a**) Percentage of spontaneous alteration behavior of experimental mice in the Y-maze. (**b**) Average escape latency to reach the hidden platform until day 5. (**c**) Time spent by the mice in the quadrant where the platform was present in training; (**d**) represents the probe test. (**e**) Western blot analysis of Aβ and BACE-1 in the experimental animal’s hippocampus. The bands were quantified using ImageJ software and the difference is shown by their respective histogram. β-actin was used as a loading control. (**f**) Confocal microscopy of Aβ and representative histogram and DAPI staining (blue) in the hippocampus (Cornu Ammonis (CA1) and Dentate Gyrus (DG regions) of adult mice. The density values are relative to those in the control group and are expressed in arbitrary units (AU), magnification 10×, scale bar = 50 μm. The data are presented as the mean ± SEM of 4 mice/group for Western blot and 4 mice/group for confocal microscopy, and are representative of three independent experiments. ω indicates a significant difference from saline-injected control mice; σ indicates a significant difference from Aβ_1–42_-injected mice. Significance = ω *p* < 0.05, σ *p* < 0.05.

We check the effects of caffeic acid against the memory dysfunctions associated with Alzheimer’s disease (AD). We performed the Y-maze and Morris water maze (MWM) tests, as discussed in the methods section. In Y-maze, the mice were placed in the center of the arms and allowed to explore the maze. As expected, control mice entered more frequently into and spent more time in the novel arm (a previously unvisited arm of the maze), while the Aβ-injected mice showed no preference for the novel arm and entered randomly into the arms and spent approximately the same amount of time in each arm, showing the deficit in spatial memory of the Aβ-injected mice. The percentage of spontaneous alternation behavior improved after caffeic acid treatment, which is an interesting sign for improved spatial working memory (Figure 2a).

Further, we performed the MWM test, where the animal is subjected to find a hidden platform to escape from the water. To perform this task, the mice form a “spatial orientation map” in the brain. During the training period, the mice were allowed to locate the hidden platform, which showed that AD mice took more time as compared to the control mice to reach the hidden platform, while treatment with caffeic acid reduced the time (Figure 2b). In the probe test, as expected, the latency to reach the position of the platform and the number of crosses over the hidden platform was reduced in the Aβ-injected mice. Caffeic acid treatment in AD mice enhanced the time in the target quadrant and crossing over the platform (Figure 2c,d). The swimming speed was not altered among the groups, so the locomotion of the mice did not affect the above mentioned parameters. Both behavioral studies suggested that caffeic acid has rescuing effects against the cognitive dysfunctions in the Aβ-injected mice brains. 

Amyloid beta (Aβ) is the main pathological hallmark of AD. We examined the expression of Aβ and beta-site amyloid precursor protein-cleaving enzyme-1 (BACE-1) in the brains of the experimental mice to examine the effects of caffeic acid against the amyloidogenic factors. The western blot results showed there was increased expression of Aβ and BACE-1 in the Aβ-induced AD mice brains compared to the wild-type saline-treated control mice. On the other hand, the administration of caffeic acid significantly reduced the expression of Aβ and BACE-1 compared to the Aβ-injected mice (Figure 2e). Furthermore, the confocal microscopic results showed increased immunoreactivity of Aβ in the AD mice brain compared to saline-treated control mice. Caffeic acid treatment reduced the expression of Aβ as compared to the AD mice (Figure 2f).

### 3.2. Neuroprotective Properties of Caffeic Acid against Aβ-Induced Oxidative Stress in the Mice Brain

**Figure 3:**
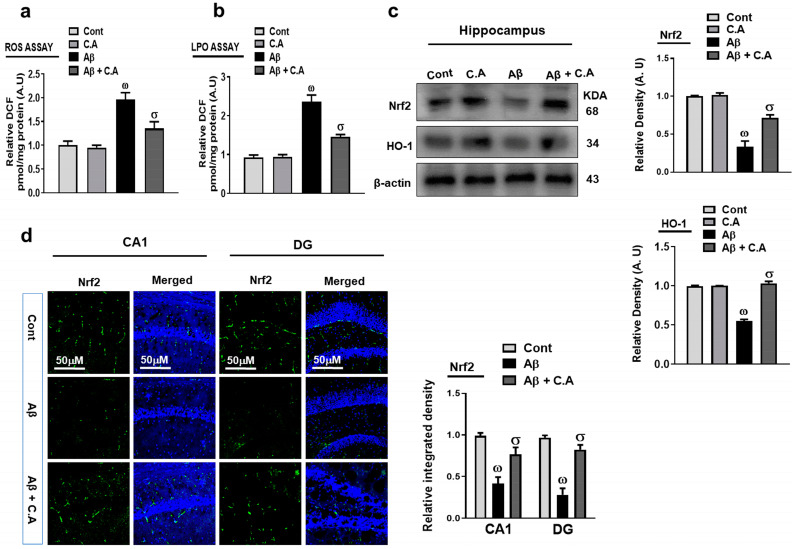
Effects of caffeic acid on oxidative stress. (**a**,**b**) Representative histograms showing ROS and LPO in the experimental mice brain hippocampus. (**c**) Analysis of the protein expression in Nrf2 and HO-1 by immunoblotting in the mouse hippocampus. Using ImageJ software, the bands were quantified, and the difference is displayed by the histogram for each band. β-actin was used as a loading control. (**d**) Immunofluorescence of Nrf2 and representative histogram and DAPI staining (blue) in the hippocampus (Cornu Ammonis (CA1) and Dentate Gyrus (DG regions) of adult mice. The density values are relative to those in the control group and are expressed in arbitrary units (AU), magnification 10×, scale bar = 50 μm. The data are presented as the mean ± SEM of 4 mice/group for Western blot and 4 mice/group for confocal microscopy and are representative of three independent experiments. ω indicates a significant difference from saline-injected control mice; σ indicates a significant difference from Aβ_1–42_-injected mice. Significance = ω *p* < 0.05, σ *p* < 0.05.

Oxidative damage has been a core factor in the pathogenesis of a number of neurodegenerative diseases, and particularly it is linked to the etiology of AD [17]. Caffeic acid is a strong antioxidant so we also examined the antioxidative properties of caffeic acid in Aβ-induced oxidative stress in the different experiments as described. First of all, we evaluated the level of reactive oxygen species (ROS) in mice brains, which showed that the intracellular ROS was increased after Aβ-injection, and treatment with caffeic acid decreased the ROS, showing that caffeic acid could reduce Aβ-induced upregulation of ROS (Figure 3a). Additionally, the lipid peroxidation (LPO) assay showed enhanced LPO in the Aβ-injected mice brains and was reduced with the administration of caffeic acid (Figure 3b). Nrf2, as an endogenous oxidative stress regulator, becomes suppressed when the ROS level is increased. We checked the expression of Nrf2 and its downstream target, heme oxygenase 1 (HO-1). Our western blot results suggested that Aβ suppressed the expression of Nrf2 and HO-1, and were reversed with the administration of caffeic acid (Figure 3c). We further confirmed our results through immunofluorescence analysis, which showed reduced expression of Nrf2 in the Aβ-injected mice brains, as compared to saline-treated control mice, and was upregulated in the caffeic acid-treated mice brains (Figure 3d).

### 3.3. Effects of Caffeic Acid against Aβ-Induced Activated Microglia and Astrocytes

**Figure 4:**
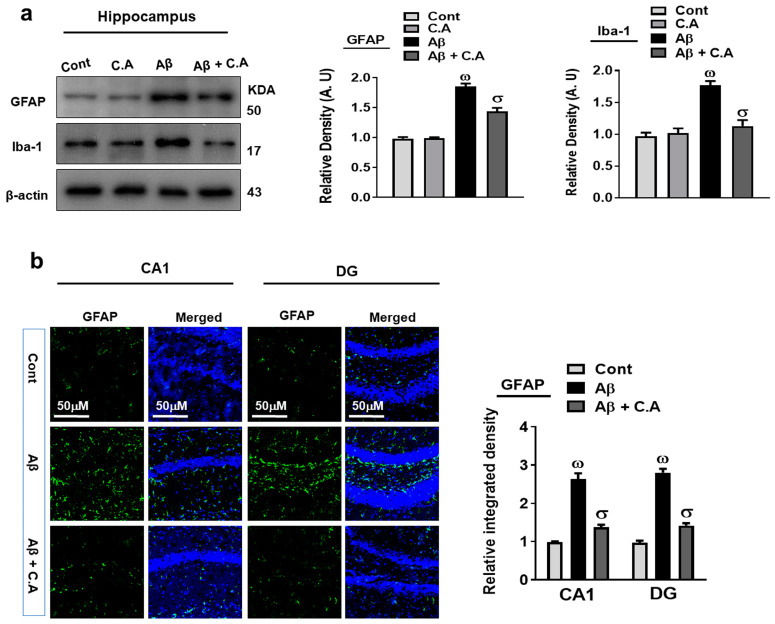
Caffeic acid reduced the activation of glial cells. (**a**) Western blot examination of GFAP and Iba-1 of mice’s hippocampus. The bands were quantified using ImageJ software and the difference showed by their respective histogram. β-actin was used as a loading control. (**b**) Confocal microscopy analysis of GFAP (green) and representative histogram and DAPI staining (blue) in the hippocampus of adult mice. The density values are relative to those in the control group and are expressed in arbitrary units (AU), magnification 10×, scale bar = 50 μm. The data are presented as the mean ± SEM of 4 mice/group for Western blot and 4 mice/group for confocal microscopy, and are representative of three independent experiments. ω indicates a significant difference from saline-injected control mice; σ indicates a significant difference from Aβ_1–42_-injected mice. Significance = ω *p* < 0.05, σ *p* < 0.05.

Glial cells in the brain perform important activities to maintain neuronal homeostasis. The accumulation of Aβ and oxidative stress in the brain leads to the overactivation of glial cells, which causes neuroinflammation and neurodegeneration [40,41]. To analyze the effects of caffeic acid against Aβ-induced activated glial cells, we evaluated the expressions of glial fibrillary acidic protein (GFAP), a marker of activated astrocytes, and ionized calcium-binding adaptor molecule-1 (Iba-1), a marker of activated microglia. Our western blot results showed that the expression of GFAP and Iba-1 were upregulated in the Aβ-induced AD mice’s brains, which were downregulated in the caffeic acid-treated mice hippocampus (Figure 4a). Further, we confirm the expression of GFAP, through confocal microscopy, which showed increased GFAP immunoreactivity in the AD mice hippocampus, but the immunoreactivity was reduced after the treatment with caffeic acid (Figure 4b). 

### 3.4. Caffeic Acid Reduced Aβ-Induced Inflammatory Cytokine in the AD Mice Brains

**Figure 5:**
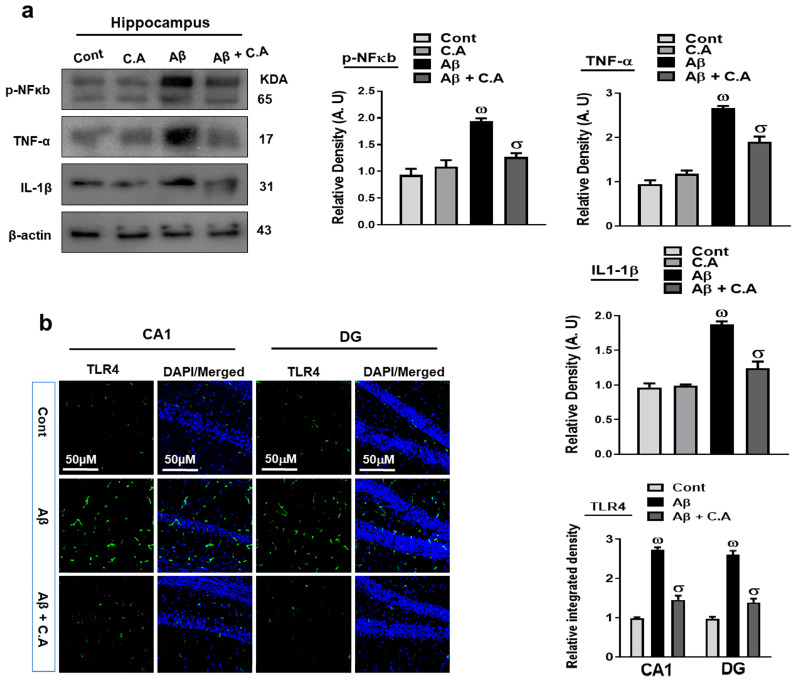
Effects of caffeic acid on inflammatory markers. (**a**) Western blot analysis of p-NFκB, TNF-α, and IL-1β in the brain of adult mice. The bands were quantified using ImageJ software and the difference showed by their respective histogram. β-actin was used as a loading control. (**b**) Immunofluorescence analysis of TLR4 (green), along with its respective histogram, and DAPI staining (blue) in the hippocampal of adult mice. The density values are relative to those in the control group and are expressed in arbitrary units (AU), magnification 10×, scale bar = 50 μm. The data are presented as the mean ± SEM of 4 mice/group for Western blot and 4 mice/group for confocal microscopy, and are representative of three independent experiments. ω indicates a significant difference from saline-injected control mice; σ indicates a significant difference from Aβ_1–42_-injected mice. Significance = ω *p* < 0.05, σ *p* < 0.05.

To unveil the anti-inflammatory effects of caffeic acid, we analyzed the expression of phosphorylated nuclear factor kappa B (p-NFkB) in the experimental groups, as p-NFkb plays a significant role in AD pathogenesis. The activated p-NFkB also releases other inflammatory cytokines, such as toll-like receptor 4 (TLR4), tumor necrosis factor-alpha (TNF-α), and interleukin 1 beta (IL-1β), which are responsible for neuroinflammation [42,43]. Our western blot results suggested an enhanced expression of p-NFkB, TNF-α, and IL-1β in the Aβ-injected mice brains, as compared to saline-injected control mice. Interestingly caffeic acid treatment in AD-induced mice significantly reduced the expression of these inflammatory cytokines (Figure 5a). We also evaluated the expression of TLR4 through immunofluorescence analysis, which showed increased immunoreactivity of TLR4 in the AD mice brain, as compared to control mice; in contrast, caffeic acid significantly reduced the expression of TLR4 in the Aβ-injected mice hippocampus (Figure 5b). The collective findings support the notion that caffeic acid may reduce Aβ-induced neuroinflammation. 

### 3.5. Effects of Caffeic Acid on PI3k/AKT, and BDNF, in Aβ-Injected Mice Brains

The phosphatidylinositol 3-kinase (PI3K)/protein kinase b (AKT) signaling pathway plays an important role in cell survival and proliferation [44]. It is reported that natural products that activate PI3K/AKT signal pathways protect the neurons from neurodegenerative disease [45]. Brain-derived neurotrophic factor (BDNF) is a neurotrophin in the CNS (central nervous system), and its activation protects the neurons against Aβ-induced neurotoxicity [46]. 

**Figure 6:**
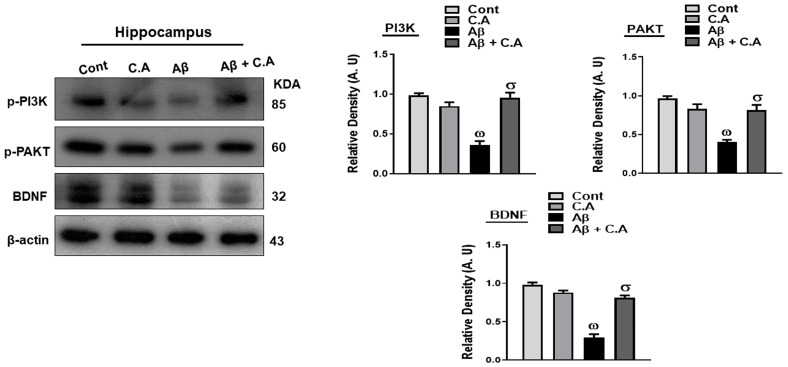
Effects of caffeic acid on p-PI3k/p-AKT and BDNF in Aβ-injected mice brains. Western blot analysis of p-PI3k, p-AKT, and BDNF, in different experimental mice groups. Using ImageJ software, the bands were quantified and the difference is shown by their respective histogram. β-actin was used as a loading control. The data are presented as the mean ± SEM of 4 mice/group for Western blot and are representative of three independent experiments. ω indicates a significant difference from saline-injected control mice; σ indicates a significant difference from Aβ_1–42_-injected mice. Significance = ω *p* < 0.05, σ *p* < 0.05.

Considering the growth-regulating effects of caffeic acid, we analyzed the expressions of PI3k/AKT signaling pathways and BDNF in the experimental mice brains. As expected, the expressions of these markers were reduced significantly in the hippocampus of Aβ-induced AD mice, as compared to saline-treated mice. Treatment of caffeic acid in AD mice significantly upregulated the expression of these markers (Figure 6).

### 3.6. Caffeic Acid Improved Synaptic Plasticity in AD-Disease Mouse Hippocampus

**Figure 7:**
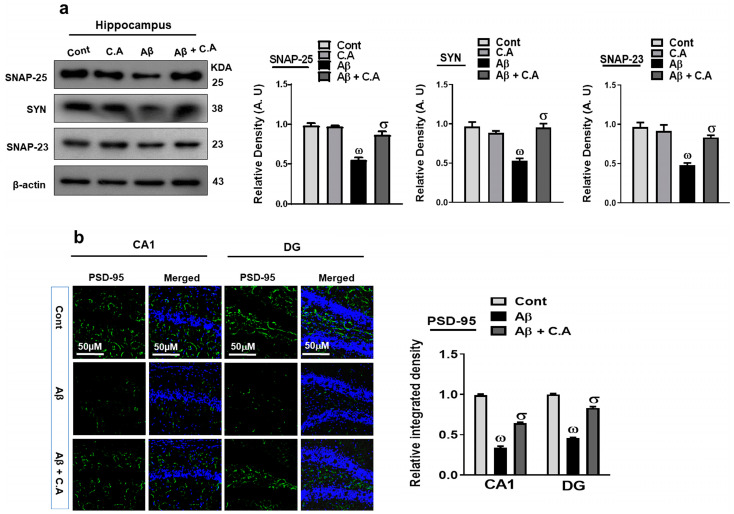
Neuroprotective properties of caffeic acid on synaptic protein expression. (**a**) Western blot analysis of synaptic proteins synaptosomal-associated protein 25 (SNAP-25) synaptophysin, and SNAP-23 proteins. ImageJ software was used to quantify the bands, and the difference is shown by their respective histogram. β-actin was used as a loading control. (**b**) Immunofluorescence analysis of postsynaptic density protein-95 (PSD-95) (green), along with its respective histogram, and DAPI staining (blue) in the brain (hippocampus). The density values are relative to those in the control group and are expressed in arbitrary units (AU), magnification 10×, scale bar = 50 μm. The data are presented as the mean ± SEM of 4 mice/group for Western blot and 4 mice/group for confocal microscopy, and are representative of three independent experiments. ω indicates a significant difference from saline-injected control mice; σ indicates a significant difference from Aβ_1–42_-injected mice. Significance = ω *p* < 0.05, σ *p* < 0.05.

Aβ is known to disrupt the synaptic function, which leads to cognitive decline and memory dysfunction [17]. To explore the effects of caffeic acid on synaptic dysfunction, we examined the levels of presynaptic (synaptosomal-associated protein 25 (SNAP-25) and synaptophysin (SYN)) and postsynaptic (SNAP-23 and postsynaptic density protein-95 (PSD-95)) proteins. Our western blot results showed decreased expression of the SNAP-25, SYN, and SNAP-23 proteins level in the AD-mouse hippocampus, as compared to control saline-treated mice. Treatment with caffeic acid increased the expression of these synaptic proteins (Figure 7a). Furthermore, we performed confocal microscopy for PSD-95, which showed decreased immunoreactivity in the AD-mouse hippocampus, as compared to control vehicle-treated mice, and treatment with caffeic acid increased the immunoreactivity (Figure 7b). 

## 4. Discussion

The root cause of Alzheimer’s disease (AD), the most prevalent neurodegenerative disease, is poorly understood, and new therapeutic strategies are urgently required. In the present study, we showed the effects of caffeic acid against the Aβ-induced AD mouse model. Our findings suggested that caffeic acid reduced the elevated oxidative stress and neuroinflammation, and improved the synaptic/memory dysfunctions in AD mice. A recent study showed that caffeic acid at 50 mg/kg prevented the mice brain from iron-overloaded toxic effects [47]. Similarly, caffeic acid (50 mg/kg) attenuated both early and delayed brain injury after focal cerebral ischemia in rats [48]. In line with these neuroprotective properties of caffeic acid, our results also suggested antiamyloidogenic effects in the brains of AD mice. 

We also targeted other cardinal features of AD, such as oxidative stress, neuroinflammation, growth factors, and synaptic dysfunction. Normally, oxidative stress is produced due to an imbalanced redox state, involving either excessive production of reactive oxygen species (ROS) or impairment in the endogenous ROS regulators [49]. The antioxidative properties of caffeic acid has been studied by several research groups. For instance, Chang-Ho Jeong et al. showed that caffeic acid has antioxidative and neuroprotective effects on neuronal cells [50]. Caffeic acid treatment enhanced antioxidant enzyme activities in C57BL/KsJ-db/db mice and prevented hyperglycemic conditions [51]. Our results also showed that caffeic acid has strong antioxidative properties and prevents the mice brain from Aβ-induced oxidative stress. Next, we examined the anti-inflammatory effects of caffeic acids against Aβ-induced neuroinflammation. Our findings suggested that caffeic acid significantly reduced the activated microglia and astrocytes in the brains of AD mice. It is reported that caffeic acid alleviates inflammatory response in rheumatoid arthritis by repressing IL-6 and TNF-α [52]. In another study, caffeic acid showed anticoagulatory, antioxidative, and anti-inflammatory properties in cardiac tissue of diabetic mice [53]. We also checked the effects of caffeic acid against Aβ-induced inflammation, which showed that caffeic acid treatment reduced the elevated expression of inflammatory mediators in the AD mouse model.

The phosphatidylinositol 3-kinase (PI3K)/protein kinase b (AKT) signaling pathway is associated with the development of several degenerative conditions, having pivotal functions in the management of oxidative damage, apoptotic cell death, neuroinflammation, and proliferation of cells [54]. Similarly, downregulation of neuronal growth factors, such as brain-derived neurotrophic factor (BDNF), plays a key role in memory dysfunctions [55], and upregulation of these factors has shown promising rescuing effects against neurodegenerative diseases [56,57]. Concerning our interest, caffeic acid markedly upregulated the expression of these markers in the brains of Aβ-injected mice. Synaptic plasticity is a key factor responsible for memory formation, and synaptic loss and dysfunction are primary features of AD [58]. In the present study, we found decreased expression of synaptic proteins in the AD mice and a significant improvement was observed with caffeic acid treatment. 

As assessed by several behavior studies, memory and cognitive functions in neurodegenerative animals were improved with caffeic acid treatment [59,60,61]. Our behavior studies also showed improved memory and cognitive functions in the caffeic acid-treated AD mice. So far, as our findings are concerned, we suggest that caffeic acid has strong antioxidant, anti-inflammatory, and growth factor regulating effects against AD-associated neurodegeneration and cognitive dysfunction. Further studies are recommended to explore the exact mechanism of action of caffeic acid against these diseases.

## Figures and Tables

**Table 1:** List of primary antibodies.

Protein Targets	Host	Application/Dilution	Catalog Number	Manufacturer
Nrf2	Mouse	WB/IF 1:1000/1:100	SC 365949	Santa Cruz Biotechnology
HO-1	Mouse	WB 1:1000	SC 136961	Santa Cruz Biotechnology
Aβ	Mouse	WB/IF 1:1000	SC 28365	Santa Cruz Biotechnology
BACE1	Mouse	WB 1:1000	SC 33711	Santa Cruz Biotechnology
Iba-1	Mouse	WB 1:1000	SC 398406	Santa Cruz Biotechnology
p-NfKB-p65	Mouse	WB 1:1000	SC 136548	Santa Cruz Biotechnology
TNF-α	Mouse	WB 1:1000	SC 52746	Santa Cruz Biotechnology
TLR4	Mouse	IF 1:1000	SC 293072	Santa Cruz Biotechnology
IL-1β	Mouse	WB 1:1000	SC 32294	Santa Cruz Biotechnology
PSD-95	Mouse	IF 1:1000	SC 71933	Santa Cruz Biotechnology
SNAP-23	Mouse	WB 1:1000	SC 374215	Santa Cruz Biotechnology
Synaptophysin	Mouse	WB 1:100	SC 17750	Santa Cruz Biotechnology
p-PAKT	Mouse	WB 1:100	SC 514032	Santa Cruz Biotechnology
BDNF	Mouse	WB 1:100	SC 65514	Santa Cruz Biotechnology

## Data Availability

Data is contained within the Manuscript.
